# Transmorphic phage‐guided systemic delivery of *TNFα* gene for the treatment of human pediatric medulloblastoma

**DOI:** 10.1096/fj.202300045R

**Published:** 2023-06-18

**Authors:** Mariam Al‐Bahrani, Paladd Asavarut, Sajee Waramit, Keittisak Suwan, Amin Hajitou

**Affiliations:** ^1^ Phage Therapy Group, Department of Brain Sciences Imperial College London London UK; ^2^ Present address: Department of Medical Laboratory Sciences, Faculty of Allied Health Sciences Kuwait University Kuwait City Kuwait

**Keywords:** bacteriophage‐guided delivery, immunotherapy, medulloblastoma, targeted systemic therapy, tumor necrosis factor‐alpha (TNFα)

## Abstract

Medulloblastoma is the most common childhood brain tumor with an unfavorable prognosis and limited options of harmful treatments that are associated with devastating long‐term side effects. Therefore, the development of safe, noninvasive, and effective therapeutic approaches is required to save the quality of life of young medulloblastoma survivors. We postulated that therapeutic targeting is a solution. Thus, we used a recently designed tumor‐targeted bacteriophage (phage)‐derived particle, named transmorphic phage/AAV, TPA, to deliver a transgene expressing the tumor necrosis factor‐alpha (*TNFα*) for targeted systemic therapy of medulloblastoma. This vector was engineered to display the double‐cyclic RGD4C ligand to selectively target tumors after intravenous administration. Furthermore, the lack of native phage tropism in mammalian cells warrants safe and selective systemic delivery to the tumor microenvironment. In vitro RGD4C.TPA.*TNFα* treatment of human medulloblastoma cells generated efficient and selective *TNFα* expression, subsequently triggering cell death. Combination with the chemotherapeutic drug cisplatin used clinically against medulloblastoma resulted in augmented effect through the enhancement of *TNFα* gene expression. Systemic administration of RGD4C.TPA.*TNFα* to mice‐bearing subcutaneous medulloblastoma xenografts resulted in selective tumor homing of these particles and consequently, targeted tumor expression of TNFα, apoptosis, and destruction of the tumor vasculature. Thus, our RGD4C.TPA.*TNFα* particle provides selective and efficient systemic delivery of *TNFα* to medulloblastoma, yielding a potential TNFα anti‐medulloblastoma therapy while sparing healthy tissues from the systemic toxicity of this cytokine.

## Graphical abstract

This study describes the efficacy of a transmorphic phage (TPA)‐guided *TNFα* gene delivery for the treatment of pediatric medulloblastoma. RGD4C peptide is displayed on the phage capsid to target αvβ3 or αvβ5 integrins, specifically overexpressed on cancer cells and tumor endothelium. RGD4C.TPA‐guided TNFα delivery resulted in cell death, which was further enhanced by the chemotherapeutic drug cisplatin (CDDP). Intravenous administration of targeted RGD4C.TPA.*TNFα* to tumor‐bearing mice resulted in selective expression of TNFα and subsequent apoptosis and destruction of tumors.

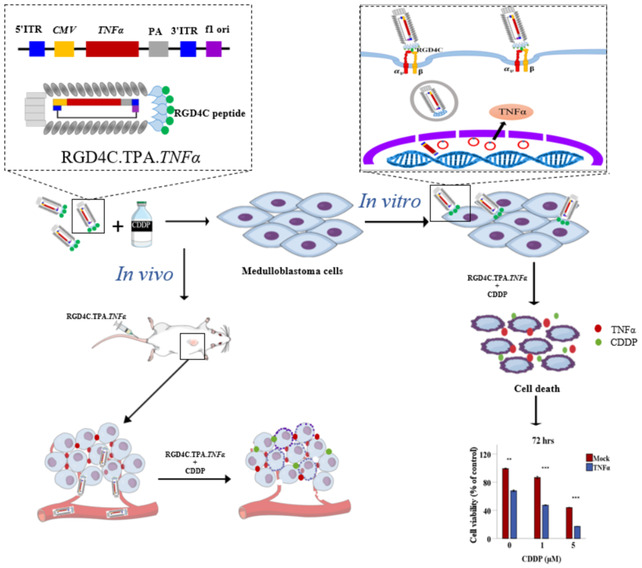

**Abbreviations**
AAV: adeno‐associated virus
AWERB: Animal Welfare and Ethical Review Body
BBB: blood–brain barrier
BSA: bovine serum albumin
CDDP: cisplatin
CMV: cytomegalovirus
DAPI: 4′,6‐diamindino‐2‐phenylindole
ECM: extracellular matrix
EDTA: ethylenediaminetetraacetic acid
ELISA: enzyme‐linked immunosorbent assay
ITR: inverted terminal repeat
PFA: paraformaldehyde
PBS: phosphate‐buffered saline
SRB: sulphorodamine B
TNFα: tumor necrosis factor‐alpha
TPA: transmorphic phage/AAV
TU: transducing units
TUNEL: terminal deoxynucleotide transferase dUTP nick end labeling

## 1 INTRODUCTION

Medulloblastoma is the most common malignant childhood brain tumor causing pediatric cancer‐related deaths.^1^ The current treatment regimen for medulloblastoma is surgical resection, radiotherapy, and chemotherapy. Despite this aggressive treatment strategy, the survivors suffer from clinically significant disabilities and long‐term medical complications such as endocrinological and neurocognitive deficits, as well as secondary tumors.^2^ Resistance to chemotherapy is a major obstacle due, at least in part, to the blood–brain barrier (BBB), while high doses result in toxic side effects associated with lack of tumor selectivity.^3^ Being a cerebellar tumor, the surgical resection on its own is associated with cognitive and motor deficits that are also induced by radiotherapy due to lesions in the cerebellum.^4^ Therefore, development of novel therapeutic approaches that are non–invasive, tumor‐selective, safer, cost‐effective, and efficient is urgently needed to improve the current treatment strategy, avoid the long‐term side effects, and save the quality of life of these young survivors.

TNFα is an inflammatory cytokine that has been reported for its anticancer properties through induction of necrosis in certain tumor types and apoptosis in others. Furthermore, TNFα was found to sensitize breast cancer cells to radiotherapy and chemotherapy through DNA damage and triggering necroptosis.^5^ Despite these properties, TNFα has limited clinical application due to its high systemic toxicity and short half‐life.^6^ We postulated targeted gene delivery for selective production of TNFα in the tumor microenvironment as a solution to reduce systemic toxicity and control the time window of TNFα bioavailability. We previously reported targeted delivery of TNFα to tumors in experimental mouse models of cancer and pet dogs with natural cancers.^7^, ^8^ Recently, we designed a tumor‐targeted transmorphic phage/AAV (RGD4C.TPA) gene delivery system that we propose to apply for targeted *TNFα* therapy against pediatric medulloblastoma.^9^ Cancer gene therapy offers a promising strategy by delivering therapeutic genes to the tumor microenvironment. As a tool for targeted and efficient gene delivery, bacteriophage‐based vectors have proven their application in prostate and breast cancers, soft tissue sarcomas, gliomas, and pancreatic cancer^10^, ^11^, ^12^, ^13^, ^14^ by using a hybrid phage vector, which was previously constructed by incorporating within the phage genome a mammalian transgene expression cassette flanked by inverted terminal repeats (ITRs) from the adeno‐associated virus AAV2.^10^, ^15^ Recently, we reported a new generation of phage‐based particles for targeted systemic gene delivery consisting of the packaging of a recombinant rAAV2 DNA by coat proteins of a filamentous phage, named transmorphic phage/AAV, or TPA.^9^ In the TPA particles, the phage genome was removed and an f1 origin of replication is the only remaining phage cis‐element to allow replication of the single‐stranded ITR‐flanked transgene expression cassette in bacteria, and its packaging into the phage capsid was provided through infection by a helper phage.^9^ The helper phage was modified to display the double‐cyclic RGD4C (CDCRGDCFC) ligand on its pIII minor coat proteins to specifically target tumors by binding to the $\alpha_{v}\beta_{3}$ integrin receptor, and at a lower extent to $\alpha_{v}\beta_{5}$.^16^ These integrin heterodimers are often overexpressed on tumor cells and tumor vasculature, but barely present in healthy tissues.^16^ Besides targeted vector entry through integrin binding, transcriptional targeting is another strategy to target gene expression within cancer cells. This can be accomplished by controlling transgene expression using cancer‐induced promoters such as the promoter of the glucose‐regulated protein 78 (*GRP78*), which is an inducible promoter that is activated by chemotherapy since the *GRP78* gene is associated with tumor resistance to treatment.^16^ We previously reported that RGD4C/phage‐*GRP78*‐guided gene expression is activated by the chemotherapeutic agent temozolomide (TMZ) in glioblastoma.^16^ Other promoters such as the cytomegalovirus (*CMV*) are well characterized for their high transcriptional activity, which can be further enhanced by the chemotherapeutic drug cisplatin (CDDP), used clinically to treat pediatric medulloblastoma.^17^ Therefore, combining chemotherapy with targeted gene delivery should increase expression of the therapeutic gene and reduce the dose of chemotherapeutic agents.

**FIGURE 1:**
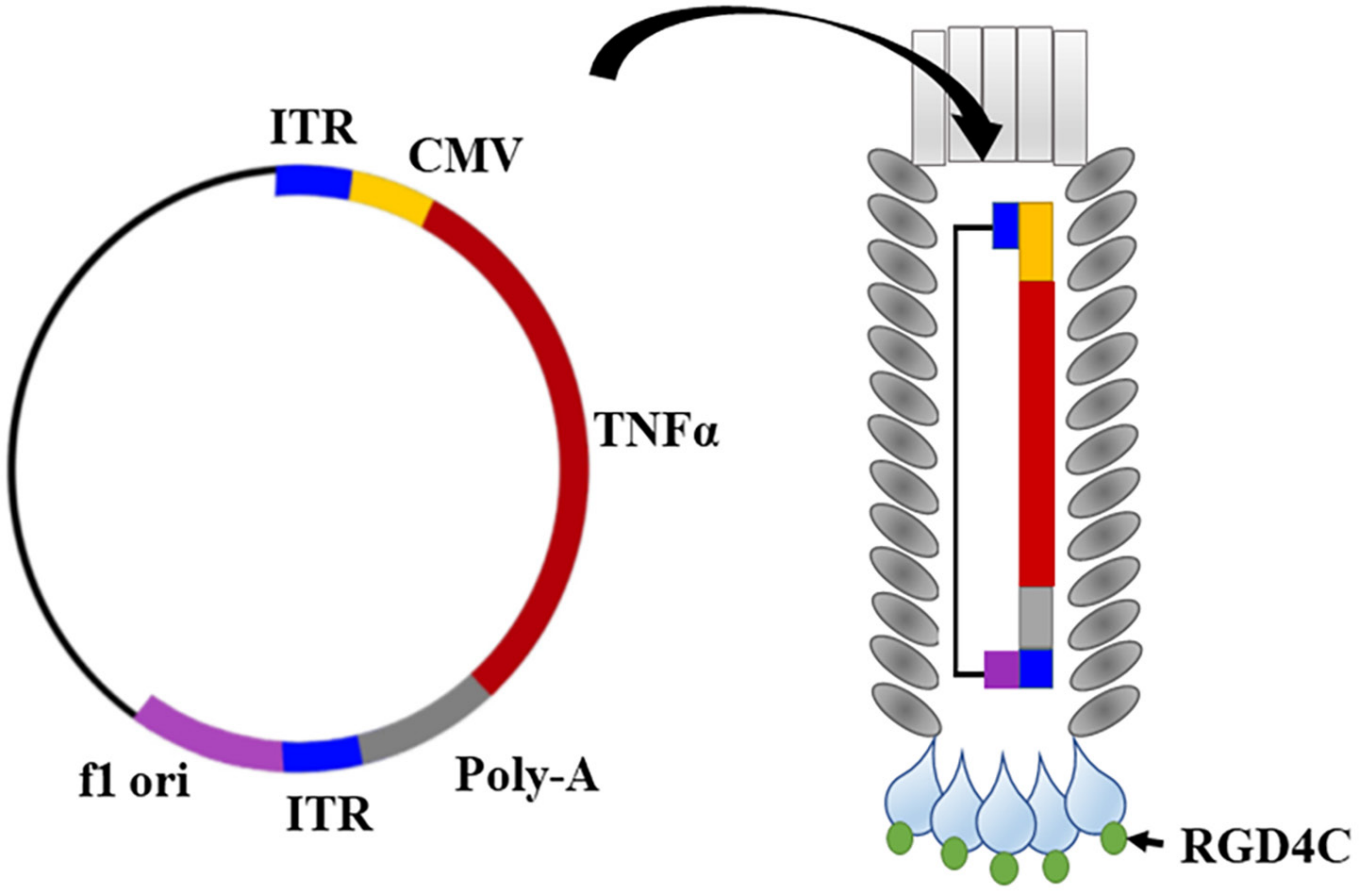
Schematic representation of targeted transmorphic phage/AAV (RGD4C.TPA) encoding the *TNFα* gene. The RGD4C ligand is displayed on the pIII minor coat proteins of the TPA capsid. A *TNFα* therapeutic DNA sequence is inserted into a mammalian transgene cassette flanked by the ITR cis‐elements, from AAV2, and downstream a *CMV* promoter. A phage origin of replication, f1 *ori*, is the only phage genetic sequence present in the TPA DNA.

In this study, we used the recently reported TPA particles characterized by enhanced gene expression, high titer production, and large DNA sequence accommodation,^9^ then constructed the RGD4C.TPA.*TNFα* (Figure 1) to evaluate the efficacy of guided systemic delivery of *TNFα* gene for medulloblastoma treatment. We investigated the efficacy of RGD4C.TPA.*TNFα* in vitro using human medulloblastoma cell lines, and in vivo in tumor‐bearing mice. We also explored targeted systemic *TNFα* therapy by RGD4C.TPA.*TNFα*, and its combination with the clinically used CDDP chemotherapeutic drug, as a strategy to enhance the therapeutic efficacy.

## 2 METHODS

### 2.1 Construction of TPA‐expressing TNFα and Lucia transgenes

TPA constructs containing the RGD4C peptide were generated by inserting the *Lucia* (secreted luciferase) reporter or therapeutic (*TNFα*) genes into the TPA plasmid under the control of the cytomegalovirus, *CMV* promoter. Briefly, *Lucia* sequence flanked by HindIII and PmlI restriction sites was digested with HindIII and PmlI restriction enzymes. While *TNFα* DNA sequence was flanked by EcoRI and BamHI restriction sites. The inserts were then ligated with the T4 DNA ligase (New England Biolabs, UK). The TPA particles were produced, as previously described,^9^ and purified from the culture supernatant of TG1 *Escherichia coli* host bacteria, then filtered through 0.45 μm filter. The TPA titer was calculated by infecting TG1 host bacteria, then colonies were counted and TPA titer was expressed as bacterial transducing units per μL (TU/μL).

### 2.2 Cell culture

The Daoy human medulloblastoma cell line was purchased from the American Type Culture Collection (*ATCC*) and UW228 cells were provided by Dr. Jonathan Ham from the Institute of Child Health, University College London, UK. The cells were cultured in D‐MEM medium containing 10% fetal bovine serum (FBS), 2 mM l‐glutamine, 100 U/mL penicillin, and 100 μg/mL streptomycin.

The cells were either authenticated by the supplier, ATCC, or by the collaborator who provided them. Upon receipt of these cell lines, they were first tested and cleared for mycoplasma contamination, then regularly tested on a monthly basis using a mycoplasma detection kit (Lonza, UK).

### 2.3 Cell transduction by TPA

UW228 and Daoy cells were seeded in multiwell plates to reach 70% confluency after 2 days. The transduction medium was prepared with TPA particles, mixed with 30 ng DEAE‐Dextran per μg of TPA, and incubated at room temperature for 15 min as we previously reported.^18^ The growth medium was replaced with the transduction medium containing either targeted RGD4C or control TPA vectors in 1% FBS, and incubated for 24 h. Next the transduction medium was removed and replaced with complete culture medium containing 10% FBS.

### 2.4 Integrin immunostaining

Medulloblastoma cells were seeded on sterile glass coverslips and allowed to grow for 2 days prior to staining. The medium was discarded, cells were washed with phosphate‐buffered saline (PBS), and fixed with 4% paraformaldehyde (PFA) at room temperature for 15 min, and then washed and treated with 50 mM ammonium chloride for 5 min. Next, cells were blocked with 2% bovine serum albumin (BSA) in PBS and 0.1% TWEEN20 at room temperature for 1 h. Primary antibodies against $\alpha_{v}$, $\beta_{3},$ and $\beta_{5}$ (Abcam, UK) were diluted 1:50 in the blocking reagent and incubated overnight. Cells were then washed and incubated with the secondary Alexa Fluor 488‐conjugated IgG antibodies (Invitrogen Life technologies), and diluted 1:750 with 0.5 μg/mL 4′,6‐diamindino‐2‐phenylindole (DAPI) for 1 h in the dark. The cells were washed and mounted with prolong gold antifade reagent (Life Technologies, UK). The samples were imaged using Zeiss LSM‐780 inverted confocal laser scanning microscope.

### 2.5 Luciferase reporter gene assay

Cells were seeded in multiwell plates and transduced with TPA‐*Lucia*. The secreted lucia was quantified in the culture medium using the QUANTI‐Luc reagent (InvivoGen, France), substrate for luciferase. Briefly, 10 μL of the medium was mixed with 25 μL of QUANTI‐Luc. Luminescence was measured using GloMax Navigator (Promega, UK).

### 2.6 Cell viability

Cells were washed with PBS and fixed with 10% trichloroacetic acid (prepared in serum‐free medium) overnight at 4°C. Fixed cells were washed with a slow running tap water and allowed to dry at room temperature, then stained with 0.4% sulphorodamine B (Sigma) for 30 min. Excess SRB dye was washed with 1% acetic acid, then cells were dried at room temperature. A 150 μL of 10 mM Tris‐base solution (pH 10.5) was added to cells and left on a shaking platform to solubilize the SRB. The absorbance was measured at 490 nm using a VersaMax Microplate Reader (Molecular Devices).

### 2.7 Quantitative real‐time PCR (qRT‐PCR)

TNFα expression at the transcriptional level was measured by real‐time qRT‐PCR using primer sequences specific to human *TNFα* and human *GAPDH*, used as reference gene. *TNFα* forward: 5′CCCAGGGACCTCTCTCTAATCA, *TNFα* reverse: 5′AGCTGCCCCTCAGCTTGAG; *GAPDH* forward: 5′CCCCTTCATTGACCTCAACTAC, *GAPDH* reverse: 5′GATGACAAGCTTCCCGTTCTC. Total RNA was extracted with TRIZOL reagent (Ambion Life Technologies, UK). Two microgram of purified RNA was treated with DNaseI (Life technologies, UK), then heat inactivated after the addition of EDTA (final concentration 5 mM) at 65°C for 10 min to stop DNaseI activity. The cDNA was generated using GeneAmp RNA PCR core kit (Applied Biosystems, UK). qPCR was performed using SYBR green universal PCR master mix (Applied Biosystems, UK) and ABI7900 real‐time PCR instrument (Applied Biosystems, UK). Gene expression was quantified using the comparative $C_{t}$ method ($∆C_{t}$) and GAPDH as the reference gene. $∆∆C_{t}$ value was calculated against reference sample of nontransduced cells. Expression was calculated as $2^{-∆∆\mathrm{CT}}$.

### 2.8 Enzyme‐linked immunosorbent assay (ELISA)

We used a human TNF∝ ELISA kit (Biolegend, UK) to evaluate expression of the TNF∝ protein in the cultured medium of cells tranduced with TPA.TNF∝. First, the ELISA plates were coated with a capture antibody diluted in a coating buffer at 1:200 dilution and incubated overnight at 4°C. Coating reagent was removed by washing five times with PBST (1X PBS with 0.05% (v/v) TWEEN20) and blocked for 1 h with 10% FBS. Then, the plates were washed, samples were added along with the standard, and incubated for 2 h with shaking. The wells were then washed, and detection antibody was added for 1 h. This was followed by the addition of Avidin‐HRP for 30 min. To minimize the background, the plates were washed and tetramethylbenzidine solution was added for 15 min in the dark. Finally, the reaction was stopped with 2N sulfuric acid and the absorbance was measured at 450 nm using VersaMax Microplate Reader (Molecular Devices).

### 2.9 Animal model and TPA‐guided gene delivery

Human subcutaneous tumors were established in SCID immune‐deficient female mice (Jackson Laboratories) by inoculation of $15\times 10^{6}$ Daoy cells into the right flank of the mice. The tumors were allowed to grow to reach a volume of ~200 mm^3^. For the homing and biodistribution experiment, TPA particles ($5\times 10^{10}$ TU/mouse) were administered twice intravenously through the tail vein 3 days apart. To quantify the TPA particles, mice were sacrificed at 18 h after the second dose, then the tumors and organs were collected for further analysis.^16^

For therapy experiments, $1\times 10^{11}$ TU/mouse TPA was injected intravenously at days 0, 4, 7, and 11. CDDP (1 mg/kg) was administered through the intraperitoneal route on days 7 and 11.

Experiments involving living mice were carried out according to the Institutional and Home Office Guidelines and under a granted Home Office–issued project license. The project license was first reviewed and approved by the Animal Welfare and Ethical Review Body (AWERB committee) at Imperial College London before its final review and approval by the Home Office.

### 2.10 Immunohistochemistry

TPA and tumor blood vessels were immunostained on 6 μm frozen tissue sections using phage‐specific and CD31 primary antibodies, respectively. The sections were first fixed in ice‐cold acetone for 10 min then washed with 0.1% PBST. Next, sections were blocked with 5% goat serum in PBST with 1% BSA for 1 h at room temperature. The sections were incubated for 48 h with rabbit anti–phage (1:500) (Sigma, UK) and rat anti‐mouse CD31 (1:50) (BD Pharmingen) primary antibodies in PBST containing 2% goat serum. This was followed by washing with PBST and staining with the secondary antibodies, AlexaFluor 488 goat anti‐rabbit IgG (1:500) and AlexaFluor 594 donkey anti‐rat IgG (1:500) (Life Technologies, UK), as well as DAPI (1:3000) in 2% goat serum, at room temperature for 1 h in the dark. Finally, the samples were washed and mounted with ProLong gold antifade reagent (Life Technologies, UK).

For the evaluation of apoptosis in tumor sections, TUNEL assay was carried out using DeadEnd Fluorometric TUNEL System kit (Promega, UK). Briefly, the sections were fixed with 4% PFA, washed, and permeabilized with 20 μg/mL proteinase K for 9 min. Then, the sections were washed with PBS, refixed with 4% PFA for 5 min, and equilibrated with equilibrium buffer for 10 min at room temperature. Next, tissue sections were labeled with terminal deoxynucleotidyl transferase (TdT) reaction mixture for 1 h at 37°C, then immersed in saline–sodium citrate (SSC) for 15 min to stop the reaction. This was followed by washing with PBS and counterstaining with DAPI (1 μg/mL) for 15 min. Finally, the tissue sections were washed and mounted with ProLong gold antifade reagent. Immunofluorescence images were acquired using Leica SP8 TCS confocal fluorescence microscope (Leica, UK).

### 2.11 Hematoxylin and eosin staining

Tumor sections were fixed with 95% ethanol for 10 min, washed with tap water, and stained with hematoxylin for 5 min. Excess hematoxylin was washed for 5 min with tap water, followed by staining with eosin for another 5 min. The sections were washed again and dehydrated in a series of increased ethanol concentrations (70%, 90%, and 100%). Finally, the sections were cleared in xylene twice, mounted in DPX‐mounting medium, and dried. The slides were examined under a light microscope (Olympus, Vanox, AHBT3).

### 2.12 Statistical analysis

Statistical analyses were performed with IBM SPSS statistics 23. Error bars indicate the standard error of the mean (SEM). Significance was determined between groups using the Student's *t*‐test and ANOVA for normally distributed data, and also Mann–Whitney and Kruskal–Wallis as nonparametric tests. *p* Values <.05 was considered statistically significant and represented as follows: *p < .05, **p ≤ .01, and ***p ≤ .001.

## 3 RESULTS

### 3.1 Human pediatric medulloblastoma cell lines express integrin cell surface receptors of RGD4C and are subsequently transduced by the RGD4C.TPA particle

**FIGURE 2:**
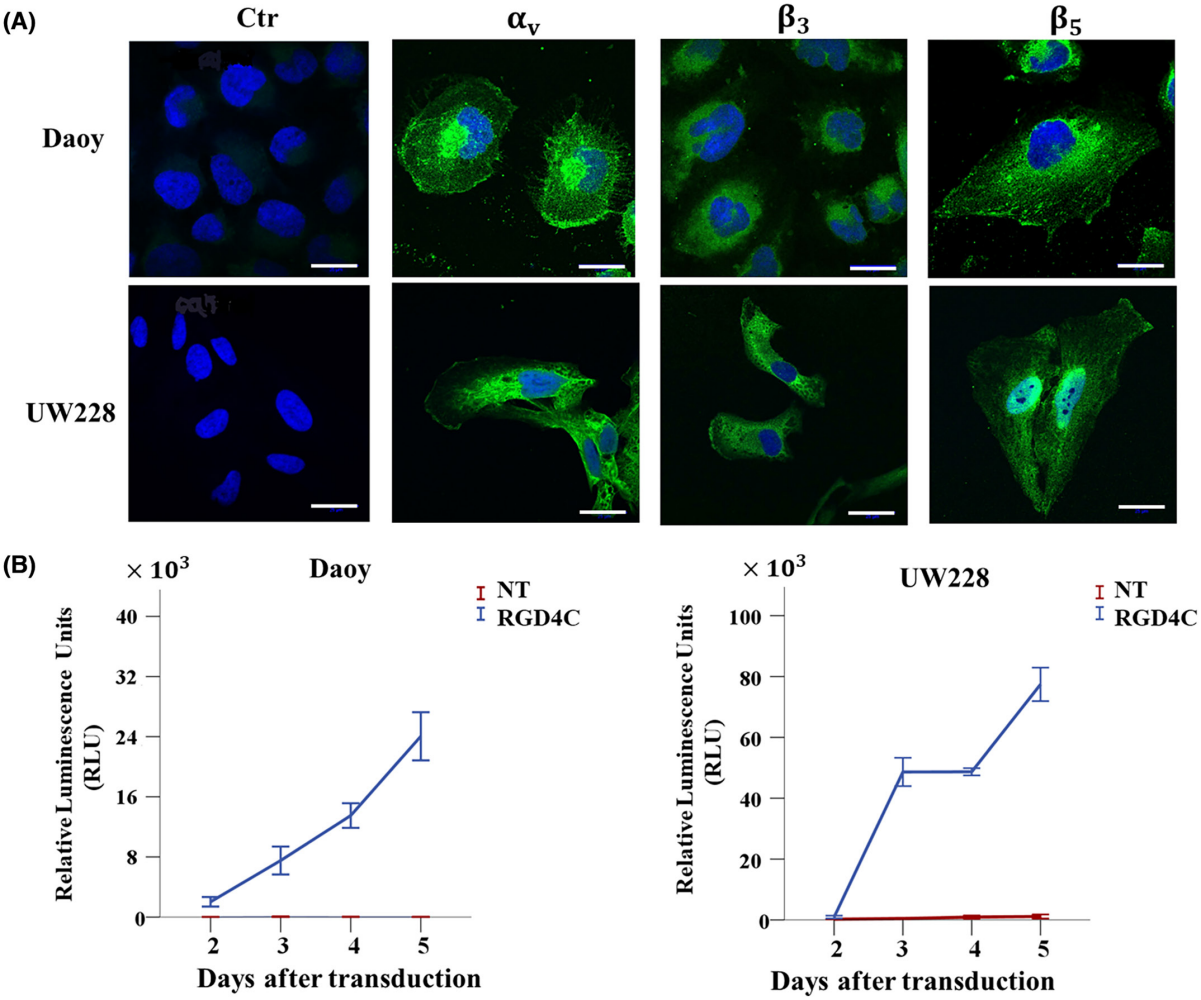
Expression of $\alpha_{v}\beta_{3}$ and $\alpha_{v}\beta_{5}$ integrin receptors in human medulloblastoma cells mediates gene delivery by the RGD4C.TPA particle. (A) Immunofluorescent staining of medulloblastoma cells was carried out without permeabilization using primary rabbit antibodies for $\alpha_{v}$, $\beta_{3},$ and $\beta_{5}$ integrins, followed by a secondary anti‐rabbit IgG AlexaFluor‐488 (Green) and 0.5 μg/mL DAPI (4′,6‐diamidino‐2‐phenylindole) (Blue). The control (Ctr) was stained with DAPI and secondary antibody alone. Scale bar, 25 μm. (B) Transduction of medulloblastoma cells with TPA.*Lucia* (targeted, RGD4C and nontargeted, NT). Secreted lucia was measured in the medium, daily, and starting from day 2 after transduction, and then expressed as relative luminescence units (RLU).

The heterodimer $\alpha_{v}\beta_{3}$ and $\alpha_{v}\beta_{5}$ integrins have been linked to cancer cell growth and metastasis.^19^ Binding of $\alpha_{v}\beta_{3}$ and $\alpha_{v}\beta_{5}$ to the extracellular matrix (ECM) proteins is known to be dependent on the presence of the RGD sequence on the ECM proteins.^20^ Thus, UW228 and Daoy medulloblastoma cell lines were investigated for integrin expression and their suitability for targeted gene delivery by the RGD4C.TPA. The expression of $\alpha_{v}$, $\beta_{3}$ and $\beta_{5}$ subunits was confirmed by immunofluorescent staining to ensure that these cells express the $\alpha_{v}\beta_{3}$ and $\alpha_{v}\beta_{5}$ integrin receptors of RGD4C. As shown in Figure 2A, both UW228 and Daoy medulloblastoma cells express all three integrin subunits.

Next, to evaluate gene delivery efficiency to medulloblastoma cells, we constructed and produced targeted RGD4C.TPA.*Lucia* carrying a reporter gene of the marine copepod‐secreted luciferase, *Lucia*. The use of secreted luciferase, lucia, is advantageous as it allows daily quantitative measurement of luciferase expression in the culture medium without lysing cells and provides higher sensitivity than intracellular luciferase.^21^ Tumor cells were transduced with targeted RGD4C.TPA.*Lucia* or nontargeted TPA.*Lucia* and expression of lucia was measured in the culture medium starting from day 2 post vector treatment (Figure 2B). A marked increase in gene expression from the RGD4C.TPA.*Lucia* was observed overtime both in Daoy and UW228 cells; in contrast, no lucia expression was detected following cell treatment with the nontargeted TPA.*Lucia* particle. These data show that the RGD4C.TPA targets human medulloblastoma cells in vitro and generates selective gene expression dependent on the RGD4C ligand.

### 3.2 RGD4C.TPA. *TNFα* targets TNFα production in medulloblastoma cells and induces tumor cell killing in vitro

**FIGURE 3:**
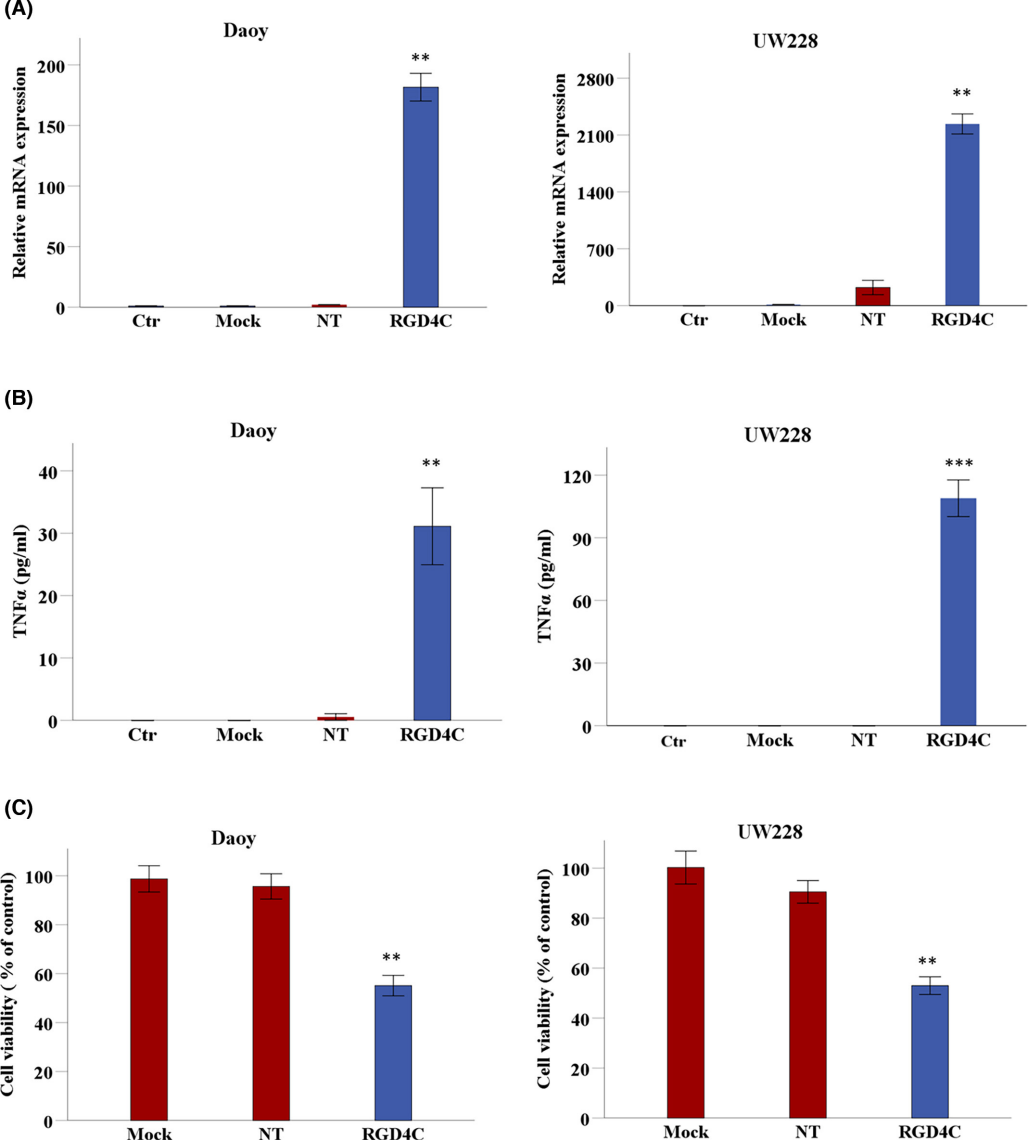
Targeted delivery of *TNFα* to medulloblastoma in vitro and assessment of the antitumor efficacy of RGD4C.TPA.*TNFα*. Daoy and UW228 cells were grown on multiwell plates, then transduced with RGD4C.TPA vector (RGD4C) carrying the *TNFα* transgene. (A) Analysis of *TNFα* mRNA expression by qRT‐PCR relative to *GAPDH* reference gene in Daoy and UW228 medulloblastoma cells at day 6 following treatment with targeted (RGD4C) or control vectors including nontargeted (NT) lacking RGD4C, and mock targeted RGD4C.TPA lacking the *TNFα* transgene (Mock). Control untreated cells (Ctr) were also included in these experiments. (B) TNF*α* protein production was measured by ELISA in the supernatant of medulloblastoma cells at day 6 post RGD4C.TPA.*TNFα* transduction. Cells treated with nontargeted or mock targeted vectors, or untreated cells were included as controls. (C) Cell viability was measured at day 6 after transduction using sulphorodamine B (SRB) assay and expressed as a percentage of viability of untreated cells. Cells treated with nontargeted or mock targeted vectors were included in this experiment as controls. Data are presented as mean ± SEM (standard error of the mean). **p ≤ .01 and ***p ≤ .001. All experiments were repeated twice in triplicate and shown are representative experiments.

After confirming the functionality of RGD4C.TPA delivery system using a reporter gene and its selectivity for the $\alpha_{v}\beta_{3}$ and $\alpha_{v}\beta_{5}$ integrins, we constructed vectors carrying a *TNFα* sequence as a therapeutic gene for medulloblastoma. To evaluate the RGD4C.TPA‐mediated delivery of *TNFα*, we carried out analysis of gene expression both at the transcriptional level by real‐time PCR and protein expression by ELISA to quantify the secreted TNFα in the media of transduced cells. Daoy and UW228 cancer cells transduced with RGD4C.TPA.*TNFα* showed high level of *TNFα* mRNA expression, which was undetectable in cells treated with the mock targeted RGD4C.TPA lacking the *TNFα* transgene, or nontargeted TPA.*TNFα* vector lacking RGD4C (Figure 3A). Then, ELISA quantification of the secreted TNFα protein in the cell culture medium showed marked TNFα production from cells transduced with RGD4C.TPA.*TNFα*, but not cells treated with nontargeted or mock targeted vectors (Figure 3B).

Next, we investigated the viability of medulloblastoma tumor cells following TPA transduction to assess the antitumor effect of RGD4C.TPA.*TNFα*. Cell death of Daoy and UW228 was significantly induced by treatment with targeted RGD4C.TPA.*TNFα* as compared to mock targeted or nontargeted vectors, which did not induce any significant tumor cell death (Figure 3C). These findings show that medulloblastoma cell killing by RGD4C.TPA.*TNFα* is selective and mediated through the RGD4C tumor‐targeting ligand.

### 3.3 Treatment with CDDP chemotherapy drug boosts TNFα expression from TPA and enhances medulloblastoma cell death

**FIGURE 4:**
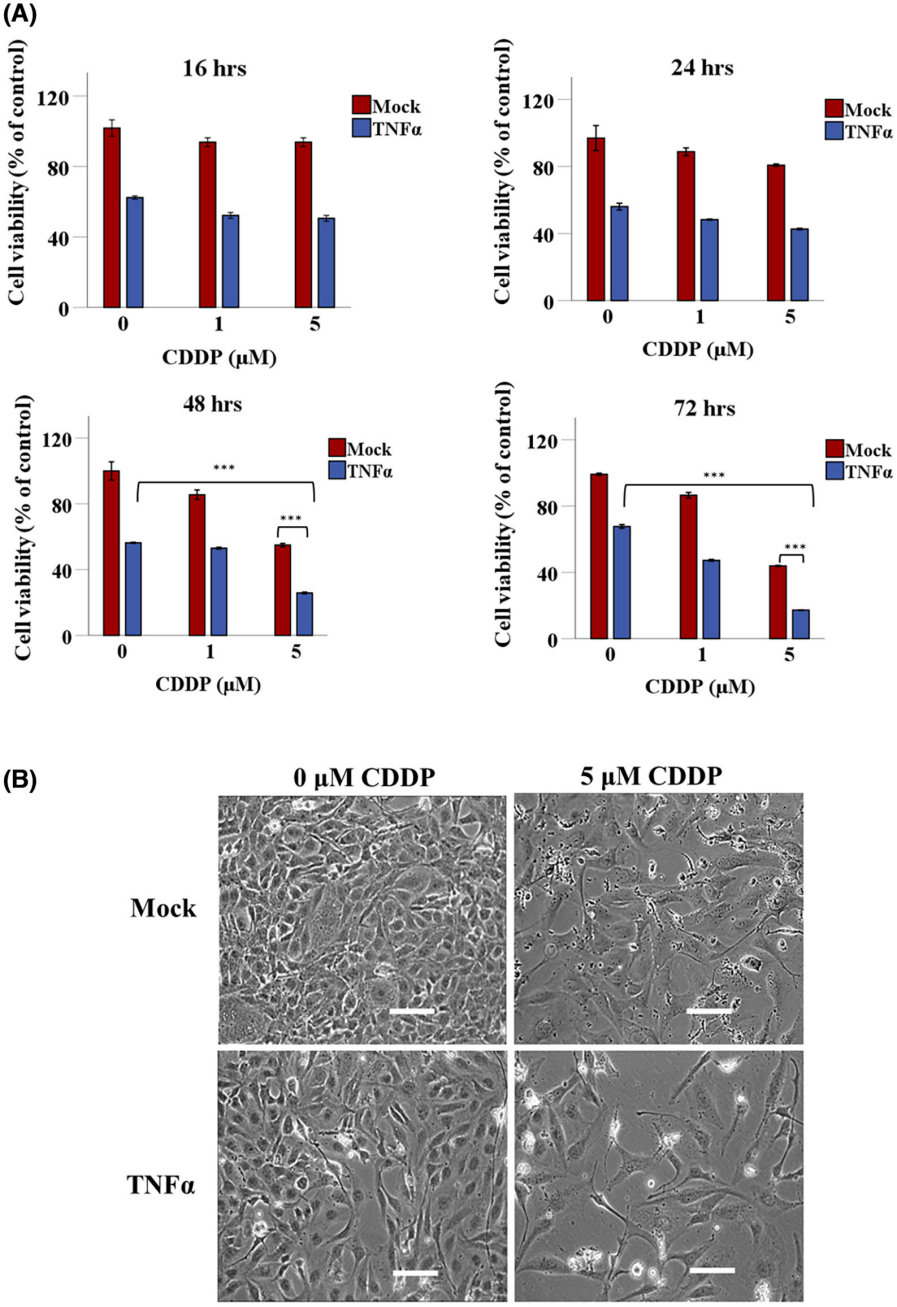
CDDP enhances TPA.*TNFα*‐mediated medulloblastoma cell death. (A) UW228 medulloblastoma cells, selected by puromycin resistance to stably carry the *GRP78.TNFα* transgene expression cassette and constitutively express TNFα, were seeded in multiwell plates and allowed to grow for 2 days, and then treated with 1 or 5 μM of CDDP. Cells carrying the *GRP78* promoter but no *TNFα* gene (mock transduction) were also included as control. Cell viability was measured at 16, 24, 48, and 72 h by using the SRB assay. (B) Representative images of stably transduced *GRP78.TNFα* tumor cells at 72 h posttreatment with 5 μM of CDDP. Scale bar, 100 μm. Data are presented as mean ± SEM (standard error of the mean). ***p ≤ .001. All experiments were repeated twice in triplicate and shown are representative experiments.

Our previous studies show that chemotherapy drugs increase targeted gene therapy by phage‐derived vectors against cancer including brain tumors.^16^, ^22^ Therefore, we sought to test the effects of combining CDDP with RGD4C.TPA.*TNFα* particle, as this chemotherapeutic agent has been used clinically to treat medulloblastoma patients.^23^ Thus, UW228 cells were transduced with RGD4C.TPA.*TNFα* under the control of the *GRP78* promoter (RGD4C.TPA.*GRP78*.*TNFα*) and selected with puromycin to generate stably transduced UW228.*GRP78*.*TNFα* tumor cells exhibiting constitutive expression of TNFα. Indeed, we previously reported that chemotherapy activates phage‐mediated gene expression under the control of the *GRP78* promoter.^16^ Cells stably transduced with a mock control TPA lacking *TNFα* were also produced. Then, UW228.*GRP78*.*TNFα* stably expressing TNFα was treated with 1 and 5 μM of CDDP and cell viability was measured over a time course at 16, 24, 48, and 72 h post CDDP administration (Figure 4A,B). CDDP treatment of UW228.*GRP78*.*TNFα* cells stably transduced with RGD4C.TPA.*GRP78*.*TNFα* induced higher cell killing compared to individual treatment with CDDP or stable transduction with RGD4C.TPA.*GRP78.TNFα* alone.

**FIGURE 5:**
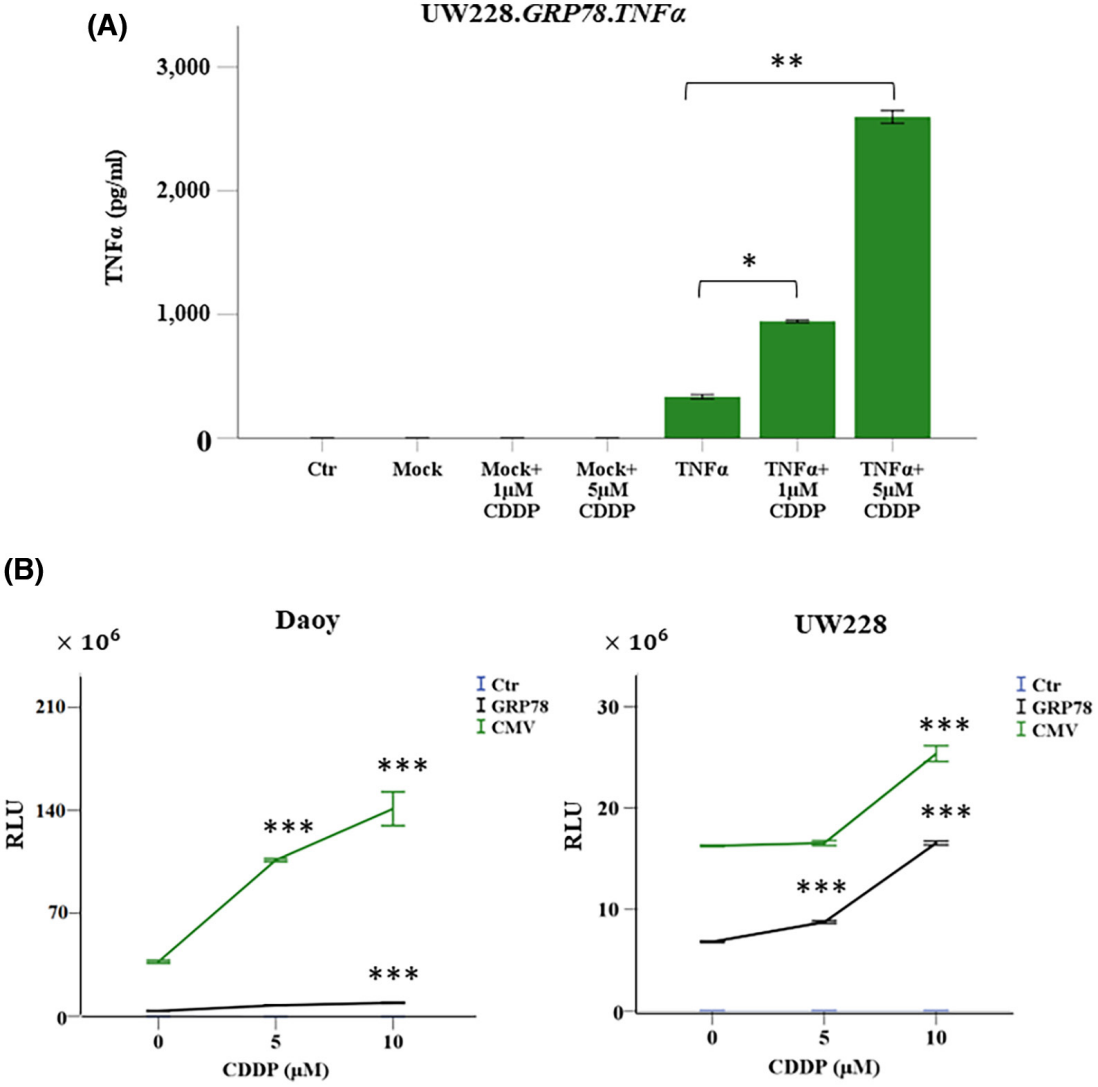
CDDP enhances gene expression under the control of *CMV* and *GRP78* promoters. (A) Quantification of TNFα expression by ELISA following CDDP treatment (1 and 5 μM) for 72 h of UW228 medulloblastoma cells transduced with TPA carrying *GRP78.TNFα* and selected with puromycin to stably express TNFα. Data were normalized to the percentage of viable control parental cells (ctr). Cells transduced with a mock vector lacking the *TNFα* transgene and treated with CDDP were also included as controls. (B) Daoy and UW228 cells were treated with RGD4C.TPA.*GRP78*.*Lucia* or RGD4C.TPA.*CMV*.*Lucia* and selected with puromycin. The cells were seeded in multiwell plates and treated with CDDP. Secreted lucia expression was measured 48 h after CDDP treatment and the results are expressed as RLU. Data are presented as mean ± SEM. *p ≤ .05, **p ≤ .01, and ***p ≤ .001. All experiments were repeated twice. Shown are data from representative experiments.

Interestingly, CDDP treatment resulted in significant induction of TNFα expression under the control of *GRP78* promoter, where 1 and 5 μM CDDP‐treated cells showed ~2‐ and ~7‐folds increase in TNFα protein expression, respectively (Figure 5A), whereas no TNFα expression was detected in cells treated with a combination of mock targeted vector and CDDP (Figure 5A). This is consistent with our previous findings that TMZ chemotherapeutic drug, clinically used for brain tumors, activates the *GRP78* promoter in human glioblastoma.^16^ Thus, before initiating in vivo studies, we sought to investigate the effect of CDDP on transgene expression by vectors carrying either *GRP78* or *CMV* promoters to select the most suitable promoter to use in efficacy studies of TPA‐guided *TNFα* expression in tumor‐bearing mice. The *CMV* and *GRP78*‐guided *Lucia* gene expression was tested using Daoy and UW228 cancer cells stably transduced with RGD4C.TPA.*CMV.Lucia* or RGD4C.TPA.*GRP78.Lucia*. Cells were then treated with two different concentrations of CDDP, 5 and 10 μM, and *Lucia* expression was measured at 48 h post chemotherapy treatment (Figure 5B). Interestingly, lucia expression in 10 μM CDDP‐treated Daoy‐*CMV‐Lucia* cells significantly increased by ~3.5‐fold. While Daoy‐*GRP78‐Lucia* showed ~2.4‐fold increase with 10 μM CDDP (Figure 5B). These results were further confirmed in CDDP‐treated UW228‐*CMV‐Lucia* and UW228‐*GRP78‐Lucia* cells, where 10 μM CDDP showed ~1.5‐fold increase in *CMV*‐guided lucia expression and ~1.8‐fold increase in *GRP78*‐guided *Lucia* expression at 48 h (Figure 5B). Therefore, based on the superiority of the *CMV* promoter in human medulloblastoma cells and its significant activation by CDDP, as compared to the *GRP78* promoter, we selected *CMV*‐guided *TNFα* expression as the sequence to use in successive in vivo investigation of RGD4C.TPA.

### 3.4 RGD4C.TPA. *TNFα* homing to medulloblastoma and biodistribution of *TNFα* expression in tumor‐bearing mice following intravenous administration

**FIGURE 6:**
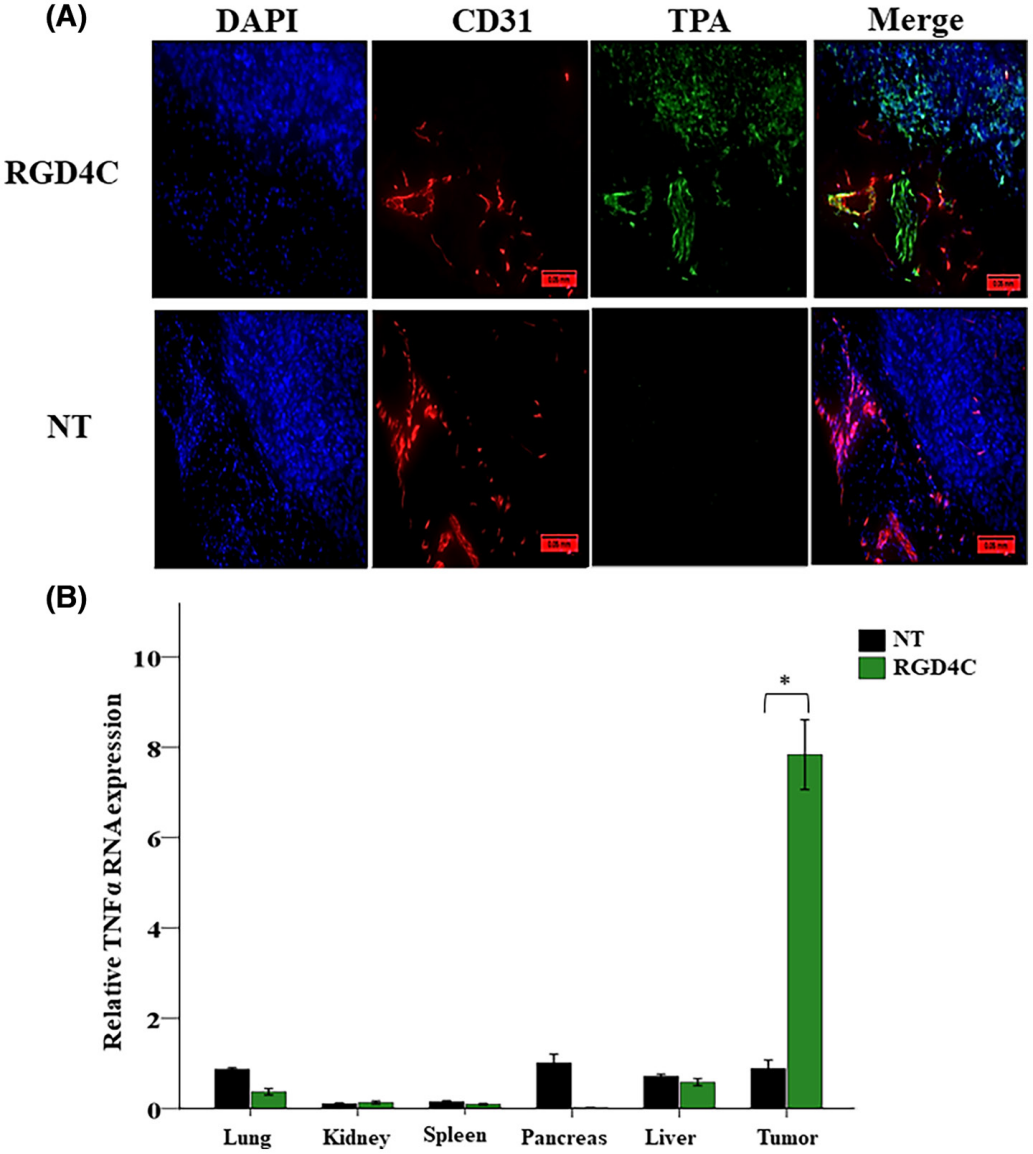
RGD4C.TPA.*TNFα* systemic targeting of medulloblastoma and biodistribution of *TNFα* expression. Daoy medulloblastoma cells were subcutaneously implanted into NOD/SCID mice and assigned randomly to either a control group TPA.*TNFα* (nontargeted, NT) or targeted RGD4C.TPA.*TNFα* group (RGD4C). (A) Tumor sections from treated mice were stained with primary antibodies for phage (green) and CD31 (red), and also with 0.5 μg/mL DAPI (blue). Scale bar, 50 μm. (B) RNA was extracted from tumors and various healthy tissues of tumor‐bearing mice injected with 5 × 10^10^ TU/mouse of targeted (RGD4C) or nontargeted TPA.*TNFα*. Expression of *TNFα* was determined by qRT‐PCR relative to *GAPDH* reference gene. Data are expressed as mean ± SEM. *p ≤ .05.

Because efficacy and safety of *TNFα* systemic therapy will depend on its selective delivery to the tumor microenvironment, initial in vivo experiments were performed to investigate RGD4C.TPA‐mediated *TNFα* gene delivery upon intravenous administration to tumor‐bearing mice. As in vivo model, we established Daoy tumors stably expressing a firefly *Luciferase* (*Luc*) reporter gene to monitor tumor growth and response to therapy by using bioluminescent imaging (BLI) of the *Luc* reporter gene. Subcutaneous tumors were established in NOD/SCID mice by implanting *Luc*‐labeled Daoy medulloblastoma cells. Next, upon detection of tumors in mice, the tumor‐bearing mice were intravenously injected with $5\times 10^{10}\;\mathrm{TU}/\text{mouse}$ of targeted RGD4C.TPA.*TNFα* or nontargeted TPA.*TNFα* vectors expressing *TNFα* under the control of *CMV* promoter. First, we assessed the tumor homing of the RGD4C.TPA.*TNFα* particles upon systemic delivery. Thus, at 18 h post vector administration, tumor‐bearing mice were sacrificed, and the tumors were harvested. To assess vector localization within the tumors, immunofluorescent staining was carried out for the phage capsid along with the blood vessel marker, CD31. The results revealed marked localization of the RGD4C.TPA.*TNFα* particles within the tumor tissue, and were detected both in the tumor vasculature and tumor cells (Figure 6A). No phage capsid staining was detected in tumors of mice following systemic treatment with the nontargeted TPA.*TNFα*.

Next, to check whether the tumor homing of RGD4C.TPA.*TNFα* translates into selective expression of TNFα in medulloblastoma, tumor‐bearing mice were intravenously injected with targeted or nontargeted TPA.*TNFα* twice at 3 days intervals. Mice were then culled at day 7 post vector delivery, and the tumors along with other major healthy tissues were harvested to perform qRT‐PCR for analysis of *TNFα* gene expression (Figure 6B). Interestingly, the data identified TNFα expression in tumors harvested from RGD4C.TPA.*TNFα*‐treated mice only, whereas no TNFα expression was detected in the lung, kidney, spleen, pancreas, and liver (Figure 6B). Additionally, no *TNFα* expression was detected in tumors or healthy tissues following intravenous administration of the nontargeted TPA.*TNFα* lacking the RGD4C ligand (Figure 6B). Together these findings support systemic tumor targeting by the RGD4C.TPA vector and its efficacy for selective TNFα gene delivery to medulloblastoma.

### 3.5 Targeted systemic therapy with RGD4C.TPA. *TNFα* against medulloblastoma in mice

**FIGURE 7:**
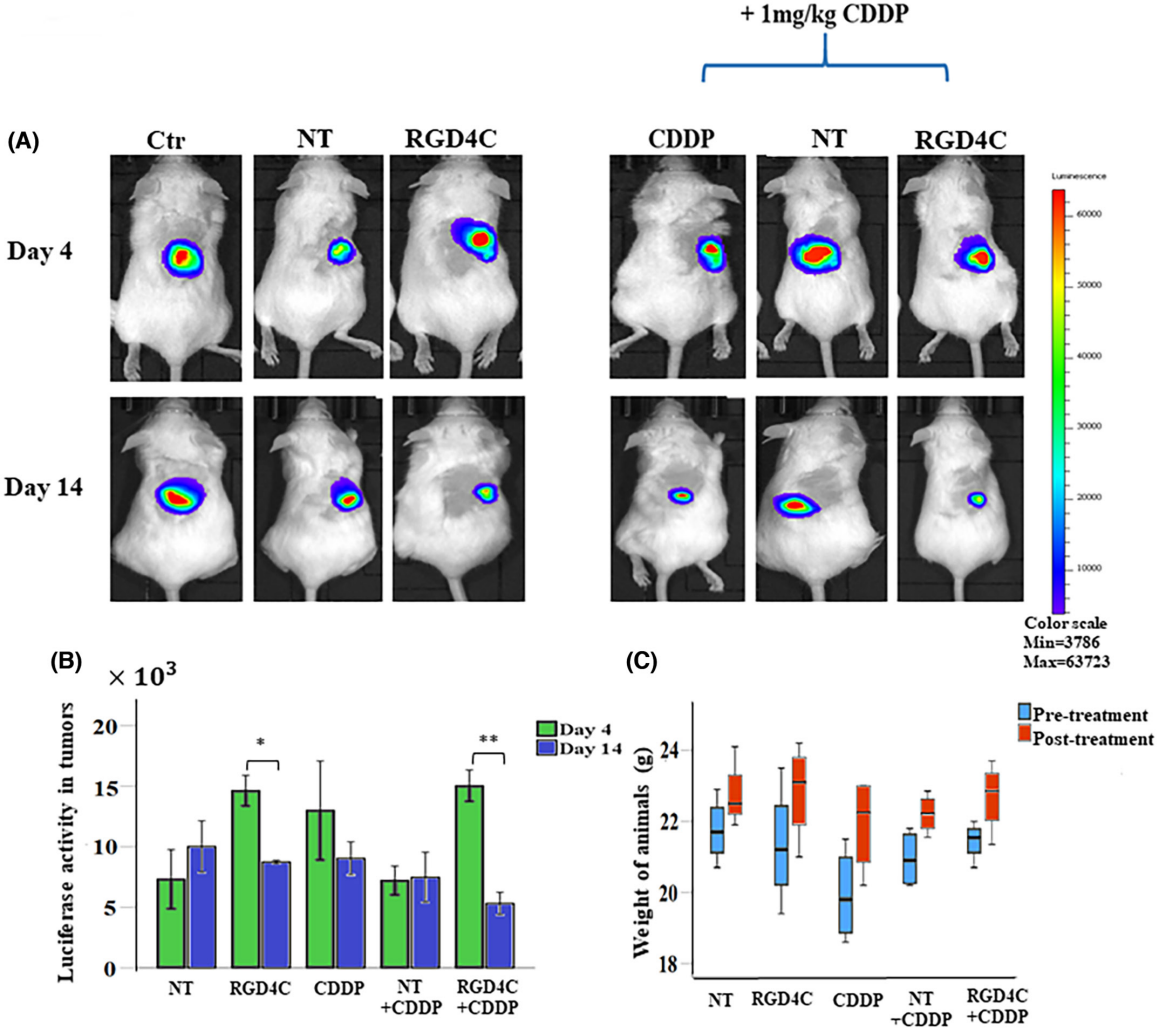
In vivo RGD4C.TPA.*TNFα* therapy in mice‐bearing subcutaneous medulloblastoma. (A) Representative tumor‐bearing mice showing in vivo bioluminescent imaging of *Luc* expression at days 4 and 14 post–treatment. Control groups (Ctr) of tumor‐bearing mice administered with saline or nontargeted (NT) TPA were also included. (B) Tumor viability measured by bioluminescent imaging (P/sec/cm^2^/sr). (C) Average animal weight during the experiment in each experimental group. The number of mice in each group is 4 (*n* = 4). Data are presented as mean ± SEM. *p ≤ .05 and **p ≤ .01.

Next, we evaluated the therapeutic efficacy of systemic delivery of RGD4C.TPA.*TNFα* against medulloblastoma in mice. Once the subcutaneous tumors reached 200 mm^3^ in size, nontargeted TPA.*TNFα* or targeted RGD4C.TPA.*TNFα* were injected intravenously twice, at days 0 and 4, via the vein tail ($1\times 10^{11}$ TU/mouse). A control group of mice injected with saline via the same route was also included. Moreover, additional groups of tumor‐bearing mice received intraperitoneal CDDP (1 mg/kg) administration along with the vector at days 0, 4, 7, 11, and 14 post vector treatment in order to investigate the effects of combination of chemotherapy and TNFα gene therapy, chemovirotherapy. This treatment regimen should allow expression of TNFα within the tumor, then administration of CDDP should induce activation of the promoter and further boost TNFα production. The tumor luminescence signals were monitored and evaluated overtime by bioluminescence imaging (Figure 7A,B). Analysis of tumor growth showed that the tumors grew larger from day 4 to day 14, post TPA treatment, in groups of mice treated either with saline or control nontargeted vector (Figure 7A), whereas mice receiving the targeted RGD4C.TPA.*TNFα* had their tumor growth reduced (Figure 7A). These data were further confirmed by the loss of tumor luminescence signal, reflecting the tumor viability, following treatment with the targeted RGD4C.TPA.*TNFα* (Figure 7B). Moreover, importantly, combination of RGD4C.TPA.*TNFα* with CDDP resulted in further antitumor effects and reduction in tumor size and tumor viability, when compared to RGD4C.TPA‐*TNFα‐* or CDDP‐treated groups (Figure 7A,B). We also monitored the animal weights during the course of therapy, and no weight loss was noticed (Figure 7C).

**FIGURE 8:**
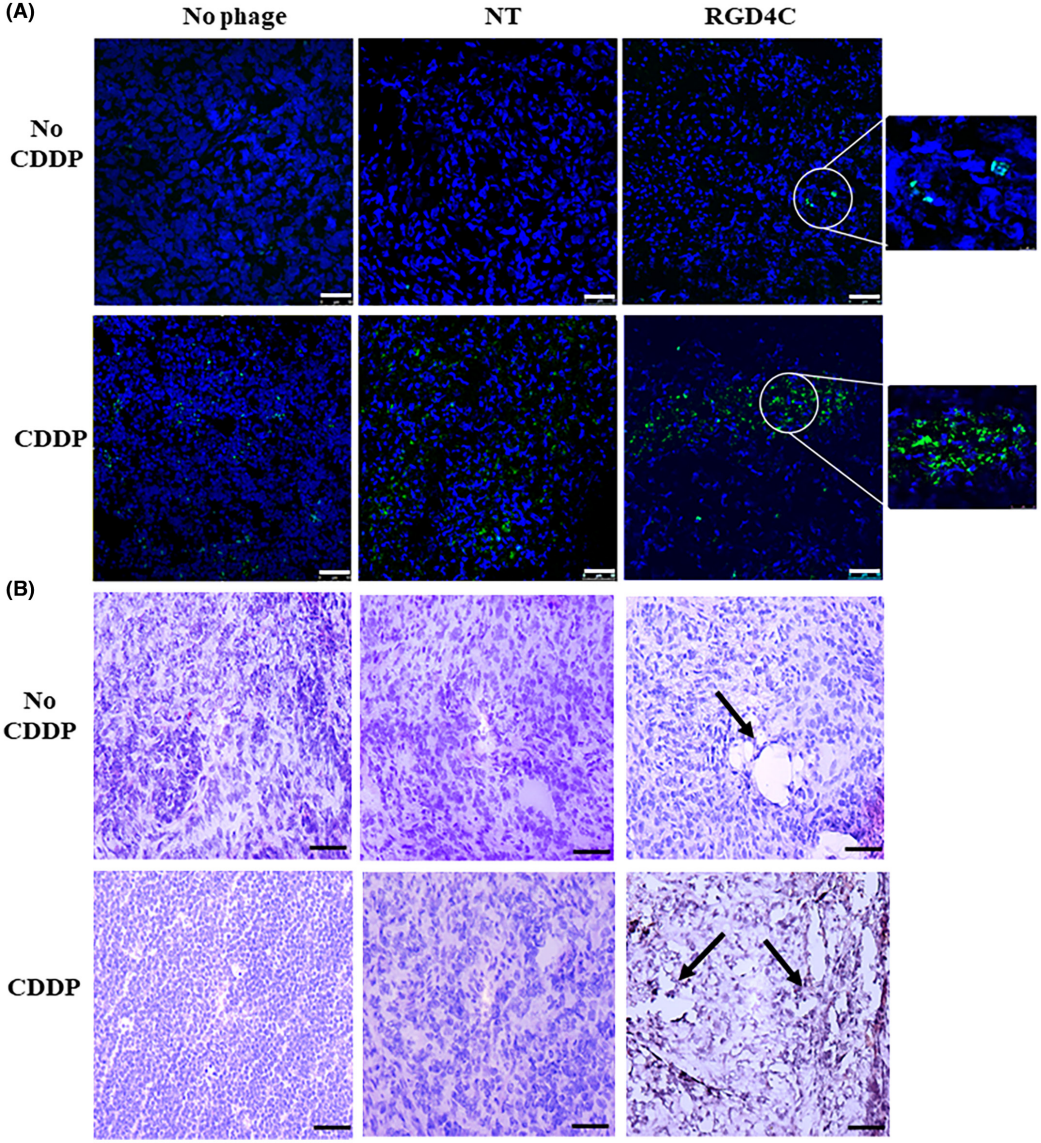
Histological analysis of tumors after therapy. (A) TUNEL assay for detection of apoptosis in tumor sections. Post–treatment tumor sections were stained using the TUNEL assay and the images were taken using a confocal microscope. DAPI staining of the sections is shown in blue. Scale bar, 50 μm. (B) Tumor sections stained with hematoxylin and eosin. Arrows indicate necrotic regions in the tumor sections. The images were taken using a light microscope. Scale bar, 100 μm.

To further confirm the antitumor effects above, we carried out a comprehensive histopathological analysis of the tumors at the end of therapy. Using the terminal deoxynucleotide transferase dUTP nick end labeling (TUNEL) assay to detect apoptotic DNA fragmentation in tumor sections, we found that combination of RGD4C.TPA.*TNFα* with CDDP induced the highest level of apoptosis compared to CDDP or RGD4C.TPA.*TNFα* alone (Figure 8A). Moreover, necrotic regions were observed in hematoxylin and eosin–stained tumor sections from mice treated with the combination treatment strategy (Figure 8B).

**FIGURE 9:**
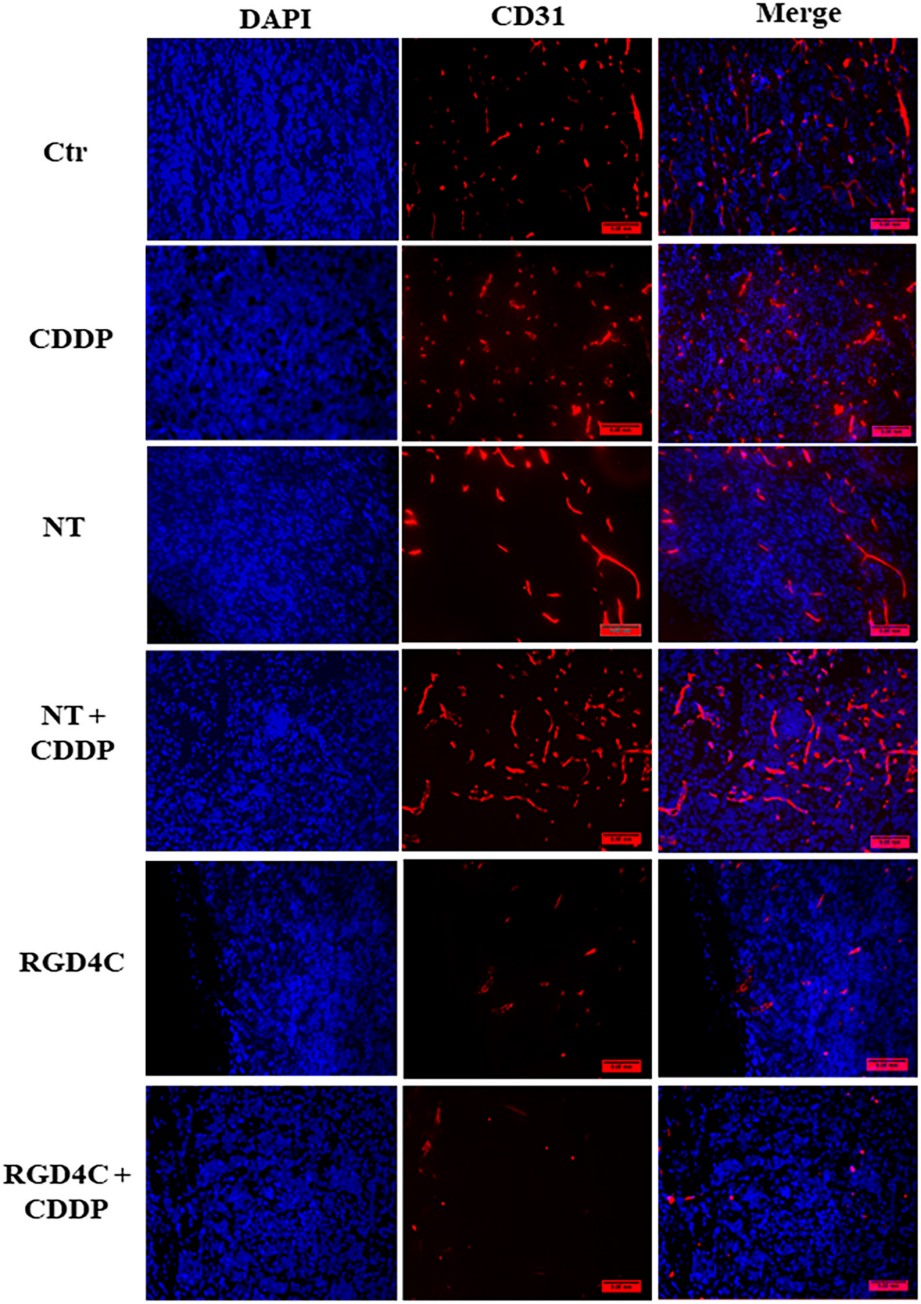
Immunohistochemistry analysis of the tumor vasculature. Tumor sections were stained with an anti‐mouse CD31 (red) as a blood vessel marker, and DAPI for nuclei staining (blue). Scale bar, 50 μm.

Moreover, the tumor sections were stained with a CD31 antibody to investigate the therapeutic effect on the tumor vasculature since the RGD4C.TPA also targets the abnormal tumor blood vessels and TNFα was reported for its antiangiogenic activity.^6^ In contrast to the nontargeted TPA.*TNFα*, where the tumor vasculature remained intact (Figure 9), the groups of mice treated with either RGD4C.TPA.*TNFα* or chemovirotherapy showed extensive vascular damage as shown by the massive reduction in the tumor vasculature (Figure 9).

## 4 DISCUSSION

In this study, we investigated the efficacy of targeted systemic delivery of *TNFα* for the treatment of medulloblastoma by using a tumor‐selective RGD4C.TPA‐mediated *TNFα* gene delivery platform. Phage‐based vectors have proven to be advantageous for delivery to brain tumors because of their ability to cross the blood–brain barrier.^16^, ^24^, ^25^, ^26^ Furthermore, the use of phage vectors is considered a safe therapeutic gene delivery strategy as they lack intrinsic tropism for mammalian cells and tissues, allowing their systemic administration.^27^ The RGD4C peptide is one of the most characterized ligands for targeting the tumor endothelium as well as tumor cells^28^; hence, our modified vector was genetically engineered to display the double‐cyclic RGD4C peptide on the phage capsid. Indeed, this peptide has been successfully used to target brain tumors such as glioblastoma.^16^, ^29^, ^30^ A large body of work has reported the tumor selectivity of RGD4C.phage‐based vectors in mice, rats, and pet dogs.^7^, ^8^, ^10^, ^16^, ^31^, ^32^ Moreover, we previously demonstrated the absence or minimal expression of the α_v_β_3_ and α_v_β_5_, integrin receptors of RGD4C in various normal human primary cells including astrocytes.^16^ Subsequently, no gene delivery was detected in these normal cells by RGD4C.phage‐derived vectors. Finally, cilengitide, which is an RGD‐derived peptide, was used clinically to treat brain tumors in cancer patients.^33^

Successful gene delivery to preclinical medulloblastoma models using mammalian viruses such as AAV has been reported to induce regression of tumor growth using Tis21 as a medulloblastoma suppressor gene; however, the direct delivery to the tumor tissue by intratumoral injections was inevitable.^1^ Thus, to avoid repeated invasive local delivery strategies, systemic administration should be less invasive and safer when both tumor targeting and lack of mammalian tropism are combined into a single delivery system.

The use of *TNFα* in this study as a therapeutic gene showed efficient antitumor activity against medulloblastoma both in vitro and in vivo upon intravenous administration. Despite its antitumor effect, the clinical application of TNFα as a therapeutic agent has had limited success due to its severe systemic toxicity.^6^ It is currently used in the clinic for the treatment of human melanoma and soft tissue sarcoma, but through local administration in the form of isolated limb perfusion.^34^ Our phage gene delivery particle is designed to deliver the TNFα cytokine into the tumor microenvironment to ensure a local and dual selective effect on both the tumor microvasculature and tumor cells. Since this delivery model targets tumor cells and tumor vasculature, the expression of *TNFα* gene is localized and self‐controlled; thus, once cell death is completed and the tumor is suppressed, *TNFα* gene expression should stop, consequently, TNFα production ceases once the therapeutic effect is accomplished. Altogether the data show that our tumor‐targeted phage delivery platform has the potential to bring systemic TNFα therapy back to the clinic to treat cancer patients via clinically noninvasive routes (e.g., intravenous).

Combining chemotherapeutic agents with gene therapy has long been used as a strategy to enhance therapeutic efficacy. Besides the additive effect of chemotherapy in inducing cell killing, it can induce gene expression through promoter activation. We previously reported such a strategy in which the chemotherapeutic agent temozolomide enhances gene expression in human glioblastoma through activation of the *GRP78* promoter.^16^ Others have shown that CDDP enhances *CMV*‐guided gene expression by inducing CMV promoter activity.^17^ Furthermore, we recently reported that the doxorubicin chemotherapeutic drug enhances phage‐guided gene expression in glioma and melanoma cancer cells by facilitating vector trafficking into the nucleus of cells.^22^ Additionally, TNFα was shown to sensitize breast cancer cells to chemotherapy, including CDDP, and subsequently enhanced the cytotoxic effect on cancer cells.^5^ TNFα was also known to permeabilize the tumor vasculature when used in the form of isolated limb perfusion through downregulation of VE‐cadherin expression.^35^ Consistent with this effect, our in vivo study showed that TPA‐guided *TNFα* delivery resulted in the destruction of tumor blood vessels; such effect should enhance the efficacy of CDDP, allowing administration of reduced doses of chemotherapy.

Together our findings support our previous studies reporting chemotherapy as an adjuvant to activate RGD4C.phage‐targeted cancer gene therapy.^16^, ^22^ Additionally, our current findings have the potential to alter the clinical use of CDDP in medulloblastoma patients and should influence targeted systemic *TNFα* therapy in this type of brain tumor. Moreover, combinatorial treatment regimens represent a promising approach to overcome chemoresistance, which is a hallmark of medulloblastoma and should allow reduction in chemotherapeutic drug administration to less toxic and cost‐effective doses.

## AUTHOR CONTRIBUTIONS

Mariam Al‐Bahrani performed the experiments and wrote the manuscript. Paladd Asavarut participated in the phage‐TNFα cloning experiments. Sajee Waramit and Keittisak Suwan contributed to the in vivo studies. Keittisak Suwan and Amin Hajitou supervised the study and participated in the experimental design. Amin Hajitou founded, designed, and supervised the whole study, then wrote and edited the manuscript.

## DISCLOSURES

The authors are inventors of patent applications describing the vector constructs reported here and will be entitled to royalties if licensing or commercialization occurs.

## Data Availability

The data that support the findings of this study are available in the methods of this article. Data sharing is not applicable to this article as no datasets were generated.
